# BeiDou Satellite Unhealthy States and the Impact on System Performance

**DOI:** 10.3390/s18124196

**Published:** 2018-11-30

**Authors:** Caibo Hu, Chuang Shi, Jinping Chen, Yidong Lou, Fei Wang

**Affiliations:** 1 GNSS Research Center, Wuhan University, Wuhan 430072, China; hucaibo@whu.edu.cn (C.H.); jinping_chen@sina.com (J.C.); ydlou@whu.edu.cn (Y.L.); 2 Beijing Satellite Navigation Center, Beijing 100094, China; wangfei_thuee@126.com

**Keywords:** BeiDou, unhealthy state, broadcast ephemeris, maneuver

## Abstract

The BeiDou system satellites may be unhealthy due to many reasons, affecting system performance in different ways. Therefore, it is important to analyze the causes and characteristics of the satellites’ unhealthy states. In this study, these states are classified into five types based on the broadcast ephemeris. Three criteria are presented, based on which a general classification method is proposed. Data from July 2017 to June 2018 are analyzed to validate the method, from which we know that the average unhealthy duration due to satellite maneuvers is much longer than the duration of unhealthy states related to satellite orbit or clock anomalies, and the other unhealthy states may be caused by inbound or outbound satellites. Statistics show that most of the time, the number of unhealthy satellites is no more than two and the average positioning accuracy in the service area will decrease by no more than 0.75 and 1.2 meters when one or two BDS satellites are unhealthy, respectively.

## 1. Introduction

Satellites will not stay in their predefined orbits all the time, since there are many unwanted perturbations in the environment [1,2]. Compared to medium Earth orbit (MEO) satellites, geostationary orbit (GEO) and inclined geosynchronous orbit (IGSO) satellites suffer more from this issue. Therefore, GEO and IGSO satellites require orbital maneuvers more frequently [3]. Unfortunately, the BeiDou System (BDS) contains many GEO and IGSO satellites [4]. Therefore, the performance of the system may be affected. In addition, there are other factors that may cause the satellites to be unhealthy. Unlike the Global Positioning System (GPS), the BDS does not provide information about planned and unplanned satellite state modifications. Therefore, it is very important to quantify the unhealthy states of BDS satellites and analyze their influence on system performance. 

The signal-in-space (SIS) performance of GPS has long been studied [5,6,7,8]. In recent years, there has been a lot of research on BDS satellite orbit and clock performance. Statistical characterization of BDS SIS errors from 2013 to 2016 were studied in [9]. Results revealed that the orbit error will be larger after satellite orbital maneuvers, resulting in larger orbit-only user range error (URE) for 2–3 days. In addition, the open service signal performance of GPS and BDS was compared [10,11]. The above research mainly focused on SIS performance when the satellites are healthy. However, unhealthy satellites will also affect the performance of the BDS by deteriorating the geometric distribution. Therefore, it is also very important to study the unhealthy state of the satellites in the navigation system.

GPS provides users with information about planned and unplanned unhealthy states of satellites via the internet, but BDS does not officially provide relevant information. Nevertheless, much research has been performed on this issue that can help us learn about the performing state of the BDS. Xie et al. presented the concept of BDS satellite individual interruption. The interruption was divided into two categories, preplanned satellite maneuvers and unplanned unhealthy states caused by abnormalities. The maneuvers were further divided into east–west and north–south station-keeping maneuvers [12]. However, a more detailed discussion was missing in the literature. Liu et al. presented a GEO satellite abnormity detection method based on wavelet analysis, and He et al. performed east–west station-keeping maneuver period extraction based on two-element and wavelet transformation [13]. However, it is difficult to choose an appropriate wavelet basis adaptively. Broadcast ephemeris can also be used to detect GEO and IGSO satellite maneuvers and anomalies. The methods can efficiently distinguish maneuvers and orbit anomalies by comparing satellite positions at the same epoch calculated with ephemerides of different reference times [14,15]. However, they cannot deal with anomalies that are not caused by orbit jumping. Huang et al. presented a maneuver and anomaly detection method by comparing the modified pseudo-range and the geometric distance calculated with broadcast ephemeris and receiver position [16,17]. This method is like the receiver autonomous integrity monitoring (RAIM) technique and can detect one satellite anomaly in real time. However, it may fail to identify faulty satellites when multiple anomalies occur. 

Existing research has mainly focused on satellite maneuvers. Satellite unhealthy states caused by other factors are not well studied and categorized, and to the authors’ knowledge their influence on BDS service performance is not known, which is the foremost concern of this paper. The main contribution of the paper is as follows. First, the BDS unhealthy states are extracted from broadcast ephemeris and divided into five categories according to the phenomena and causes. Second, the abnormal unhealthy data are wiped off and the criteria for classification are presented based on the broadcast ephemeris. In addition, a general classification method is proposed. Third, all the unhealthy state statistics from July 2017 to June 2018 are studied and several interesting phenomena are found and explained, providing us with a deeper understanding of BDS satellite unhealthy states. Last but not least, the influence of unhealthy BDS satellites on service performance is analyzed, providing a reference for BDS operation and management.

## 2. Unhealthy BDS Satellite Classification

The BDS broadcast ephemeris is updated every hour, which is twice the update frequency of the GPS. Broadcast ephemeris with higher update frequency can help us understand the satellite status better. In this section, the BDS satellite unhealthy states are extracted from the broadcast ephemeris. They are evaluated and their different causes are analyzed. These states can be divided into the following five types.

### 2.1. Type 1: Unhealthy State Due to Orbital Maneuver

Due to slight asymmetries in the Earth’s gravitational field, the gravitational pull of the Sun and the Moon, as well as some other factors such as solar radiation pressure (SRP), a satellite will drift from the intended orbit over time [18]. Therefore, a maneuver is required to maintain the satellite’s orbit. During a maneuver, a satellite will apply a thrust force to change its position and the orbit can no longer be precisely computed with the broadcast ephemeris. Consequently, the satellite state will be changed to unhealthy before a maneuver begins. A BeiDou satellite orbital maneuver usually lasts 20 minutes to 2 hours [3]. However, the unhealthy state caused by a maneuver usually lasts for more than 5 hours. This is because the data required for orbit and clock determination need several hours to accumulate and be verified. Therefore, if an unhealthy state detected in the broadcast ephemeris lasts for less than 5 hours, it is definitely not caused by a satellite maneuver.

**Figure 1:**
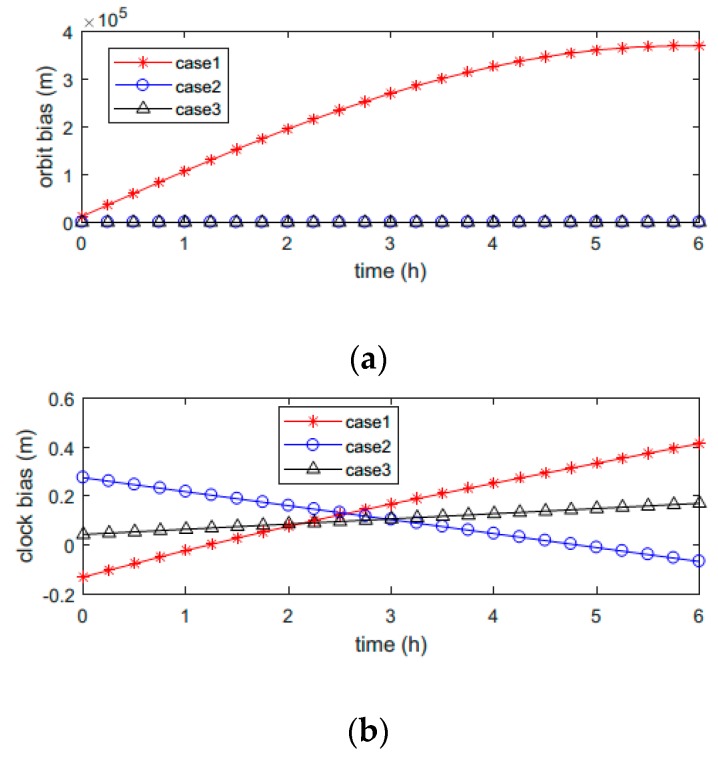
Orbit and clock biases between results calculated with ephemerides before and after the unhealthy state during the satellite unhealthy period. Different cases represent results calculated with different ephemerides. Case 1 represents a maneuvered satellite orbit and clock biases between results calculated with the last healthy broadcast ephemeris before the maneuver and the first healthy one after the maneuver. Case 2 is biases of another satellite without maneuver; parameters are the same as those in case 1. Case 3 represents the maneuvered satellite orbit and clock biases calculated with the last healthy broadcast ephemeris before the unhealthy state and the first unhealthy one after the maneuver began. (**a**) Orbit biases in different cases; (**b**) Clock biases in different cases.

It should be noted that a maneuver will cause a significant orbit change, which is much larger than the orbit prediction accuracy of the broadcast ephemeris. Consequently, it is easy to detect a maneuver by comparing the orbit results at the same epoch calculated with ephemerides before and after a maneuver. The orbit and clock biases between results calculated with ephemerides of different epochs during the satellite unhealthy period are shown in Figure 1. They correspond to the second unhealthy state of C04 on the 29th day of 2018, which was caused by a satellite maneuver. Coincidentally, two other types of unhealthy satellites also occurred on C04 on the same day, which are shown in the following.

Lines with different colors and markers represent different cases. Case 1 represents a maneuvered satellite orbit and clock biases between the results calculated with the last healthy broadcast ephemeris before the maneuver and the first healthy one after the maneuver. Since the satellite position changed significantly during the maneuver, the orbit bias was very large after the maneuver. On the other hand, the clock bias was around zero and quite stable, since the maneuver did not modify the satellite clock. Case 2 consists of biases of another satellite without maneuvering. The times of ephemerides used in this case are the same as those in case 1. This shows that the orbit bias was much smaller than that of the maneuvered satellite, because the broadcast ephemeris can provide a relatively precise orbit result in several hours. Case 3 represents the maneuvered satellite orbit and clock biases calculated with the last healthy broadcast ephemeris before the unhealthy state and the first unhealthy one after the maneuver began. Even though the broadcast ephemeris was set to unhealthy and could not reflect the position of the satellite during the maneuver, it was still updated every hour. The orbit and clock biases in case 3 were also very small, since the ephemerides were consistent, as shown in Figure 1. In other words, if only the adjacent ephemerides before and after the unhealthy state occurred are employed (case 3), the maneuver case cannot be distinguished from the non-maneuver case (case 2). It can also be inferred from the figure that all the clock correction parameters before, during, and after a maneuver can be used to get accurate clock correction results.

### 2.2. Type 2: Unhealthy State Corresponding to Excessive Orbital Error

**Figure 2:**
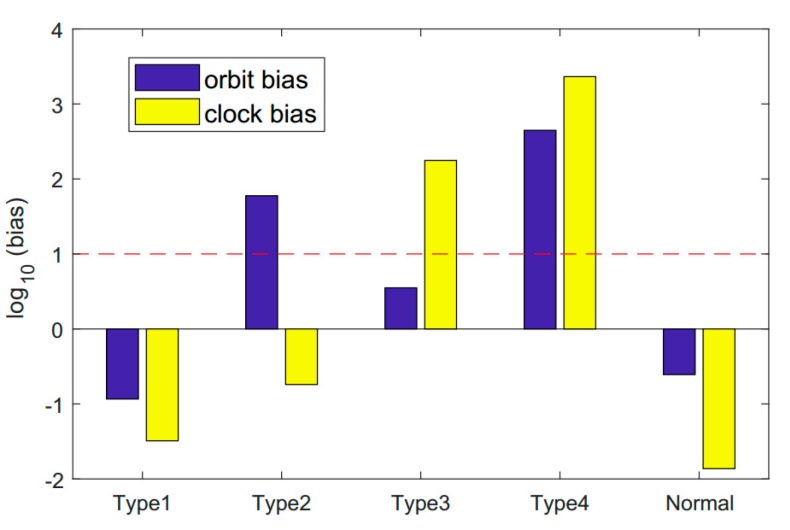
Orbit and clock biases between results calculated with the last healthy ephemeris and the first unhealthy one. Types 1, 2, 3, and 4 correspond to Section 2.1, Section 2.2, Section 2.3 and Section 2.4, respectively. A normal case in which no unhealthy state occurred is also provided for comparison.

Maneuvers can cause satellite positions to deviate from the results calculated with the broadcast ephemeris. However, there are other reasons for an excessive orbital error. For example, satellite orbital results may be inaccurate due to poor-quality observation data, which is very common, since the data may be lacking due to frequent satellite maneuvers and insufficient observation stations for the IGSO and MEO satellites. In addition, a satellite payload failure may also cause excessive orbital error, leading to a temporary unhealthy state. Orbit and clock biases between results calculated with the last healthy ephemeris and the first unhealthy one of Type 2 are shown in Figure 2.

### 2.3. Type 3: Unhealthy State Corresponding to Excessive Satellite Clock Error

Anomalies caused by excessive satellite clock error are rarely mentioned in the current literature. However, this is also a very common cause of satellite unavailability. These abnormal states can be further divided into the following two categories. First, an unexpected jump of satellite clock phase will greatly affect the navigation service, so the satellite should be set as unhealthy. Second, after a satellite payload failure occurs, the payload will be reset by the BDS control station. The satellite clock will be initialized, resulting in an excessive satellite clock jump. In fact, the BDS monitor stations can detect such failures in real time and update new clock parameters; therefore, such unhealthy states can usually be fixed in a very short time. Orbit and clock biases between results calculated with the last healthy ephemeris and the first unhealthy one of Type 3 are also provided in Figure 2.

### 2.4. Type 4: Unhealthy State Corresponding to Excessive Orbital and Clock Error

Sometimes, satellite orbital and clock malfunctions may occur simultaneously due to failures in the BDS ground control system or satellite payload, which can be viewed as a mixture of unhealthy types 2 and 3, as mentioned before, and the satellite will also be set as unhealthy until the ephemeris is corrected or the payload is recovered. 

In order to gain insight into the different types of unhealthy states, orbit and clock biases between results calculated with the last healthy broadcast ephemeris and the first unhealthy one when the unhealthy state occurred are provided in Figure 2. Type 1 is the second unhealthy state that occurred on C04 on the 29th day of 2018, corresponding to a satellite maneuver. Type 2 is the third unhealthy state that occurred on C04 on the 29th day of 2018, corresponding to an unhealthy state caused by excessive orbital error. Type 3 is the first unhealthy state that occurred on C04 on the 29th day of 2018, corresponding to an unhealthy state caused by excessive clock error. Type 4 is the unhealthy state that occurred on C03 on the 220th day of 2017, corresponding to an unhealthy state in which orbital and clock malfunctions occurred simultaneously. A normal case in which no unhealthy state occurred is also provided for comparison.

Figure 2 shows that different types of unhealthy states can be distinguished by the consecutive ephemerides before and after the unhealthy state occurs. However, an unhealthy state caused by satellite maneuvers cannot be distinguished from the normal case; these two cases can be distinguished by using the last healthy ephemeris before the unhealthy state occurred and the first healthy ephemeris after the satellite recovery.

### 2.5. Type 5: Unhealthy State Due to Inbound or Outbound Satellite

Since the BDS monitor and uploading stations are mostly located in China, there will be no observation data and the broadcast ephemeris will not be uploaded and updated when the IGSO and MEO satellites depart from the visible area of the BDS stations. Usually, these satellites will re-enter the visible area of the monitor stations after a few hours to more than 10 hours. Hereafter, we refer to satellites that leave and re-enter the visible area of the monitor station as outbound and inbound satellites, respectively. Under normal circumstances, the ephemeris is still available after the satellite re-enters the visible area. However, after a satellite maneuver or a satellite failure, the accumulated observation data will be abandoned and the orbit and clock prediction accuracy will deteriorate. As a result, the accuracy of the ephemeris may fail to meet the requirements of the system performance for an inbound satellite. Therefore, the master control station should set the satellite state to unhealthy before the satellite leaves the visible area. This unhealthy state will last for a few hours to more than 10 hours, determined by the length of time the satellite is out of the visible area. However, it can still be distinguished from the satellite maneuvers by using the broadcast ephemeris, which will be introduced in the next section.

## 3. Criteria of Satellite Unhealthy State

According to the analysis in Section 2, it can be inferred that different kinds of BDS satellite unhealthy states have different characteristics, which can be applied for unhealthy state classification. In this section, three criteria of satellite unhealthy state are presented: maneuver, orbital error, and clock error.

### 3.1. Maneuver Criterion

Reference [14] calculated the root mean square (RMS) of the difference of the mutual orbit results obtained with ephemerides of different epochs in a period of time. In fact, according to Figure 1, only the orbit difference after the satellite is healthy again is required to detect a maneuver, which is called maneuver satellite positioning index (mspi) in the paper. The criterion is defined in (1):

(1) $$mspi\left(t_{0},t_{e}\right)=\left|\left|p\left[eph\left(t_{0}\right),t_{e}\right]-p\left[eph\left(t_{e}\right),t_{e}\right]\right|\right|$$

where $t_{0}$ is the epoch of last healthy broadcast ephemeris before the satellite state is set to unhealthy and $t_{e}$ is the epoch of the first healthy broadcast ephemeris after the maneuver, ||*|| represents the Euclidean distance symbol, eph(t) is the broadcast ephemeris at epoch t, and $p[eph(t_{i}),t_{j}]$ represents the satellite position at $t_{j}$ calculated with the broadcast ephemeris at epoch $t_{i}$. The mspi satisfies the following inequality:

(2) $$\begin{array}{cc} mspi(t_{0},t_{e}) & =||p[eph(t_{0}),t_{e}]-p[eph(t_{e}),t_{e}]||\\ & =||p[eph(t_{0}),t_{e}]-p_{a}(t_{e})+p_{a}(t_{e})-p[eph(t_{e}),t_{e}]||\\ & \leq ||p[eph(t_{0}),t_{e}]-p_{a}(t_{e})||+||p_{a}(t_{e})-p[eph(t_{e}),t_{e}]|| \end{array}$$

where $p_{a}(t)$ is the actual position of the satellite at epoch t. When there is no satellite maneuver, both ephemerides at $t_{0}$ and $t_{e}$ can be used to calculate the approximate satellite positions at epoch $t_{e}$. Consequently, the mspi will be very small. When a satellite maneuver occurs, the satellite position calculated with the ephemeris at $t_{0}$ will obviously deviate from the true position at $t_{e}$. Therefore, the first term of the right side in Equation (2) will be very large and the mspi will exceed the threshold.

According to Figure 1, $mspi(t_{0},t_{e})$ is very large (usually several thousand to tens of thousands of meters) when the unhealthy state is caused by a satellite maneuver and the duration of the unhealthy state is not less than 5 hours. On the other hand, $mspi(t_{0},t_{e})$ is usually much smaller when no abnormality occurs or the unhealthy state is not caused by a maneuver. The analysis is consistent with the results in Figure 1.

### 3.2. Orbit Error Criterion

In order to detect an unhealthy state caused by excessive orbit error, the unhealthy satellite positioning index (uspi) is proposed, according to Figure 2. It is defined as follows:

(3) $$uspi\left(t_{0},t_{1}\right)=\left|\left|p\left[eph\left(t_{0}\right),t_{0}\right]-p\left[eph\left(t_{1}\right),t_{0}\right]\right|\right|$$

where $t_{1}$ is the epoch of the first unhealthy broadcast ephemeris after the satellite state is set to unhealthy. The uspi satisfies the following inequality:

(4) $$\begin{array}{cc} uspi(t_{0},t_{1}) & =||p[eph(t_{0}),t_{0}]-p[eph(t_{1}),t_{0}]||\\ & =||p[eph(t_{0}),t_{0}]-p_{a}(t_{0})+p_{a}(t_{0})-p[eph(t_{1}),t_{0}]||\\ & \leq ||p[eph(t_{0}),t_{0}]-p_{a}(t_{0})||+||p_{a}(t_{0})-p[eph(t_{1}),t_{0}]|| \end{array}$$

The uspi should be close to zero, since the broadcast ephemerides are normally consistent at adjacent epochs. When the prediction accuracy of the satellite orbit is poor, the second term of the right side in Equation (4) may be very large. Therefore, a uspi that is over the preassigned threshold indicates that the satellite is suffering from excessive orbit error, which may affect the service performance of the system. It should be noted that the uspi corresponding to an unhealthy state caused by a satellite maneuver will not exceed the threshold either. Therefore, it can be used to distinguish the unhealthy state caused by a maneuver and an excessive orbit error.

### 3.3. Clock Error Criterion

The criterion used to detect an unhealthy state caused by satellite clock error is given in Equation (5), which is called the unhealthy satellite clock index (usci):

(5) $$usci\left(t_{0},t_{1}\right)=\left|clk\left[eph\left(t_{0}\right),t_{0}\right]-clk\left[eph\left(t_{1}\right),t_{0}\right]\right|$$

where $clk[eph(t_{i}),t_{j}]$ represents the satellite clock error at $t_{j}$ calculated with the broadcast ephemeris at epoch $t_{i}$, and |*| represents the absolute value symbol. The usci satisfies the following inequality:

(6) $$\begin{array}{cc} usci(t_{0},t_{e}) & =|clk[eph(t_{0}),t_{0}]-p[eph(t_{1}),t_{0}]|\\ & =|clk[eph(t_{0}),t_{0}]-clk_{a}(t_{0})+clk_{a}(t_{0})-clk[eph(t_{1}),t_{0}]|\\ & \leq |clk[eph(t_{0}),t_{0}]-clk_{a}(t_{0})|+|clk_{a}(t_{0})-clk[eph(t_{1}),t_{0}]| \end{array}$$

When the prediction accuracy of the satellite clock is poor or the satellite suffers an accidental clock jump, the second term on the right side of Equation (6) will be very large. Therefore, the usci may be over the preassigned threshold, indicating that the satellite is suffering from excessive clock error. It should be noted that a payload failure may also cause an excessive clock error. However, a satellite maneuver cannot lead to a large usci.

### 3.4. Clock Error Criterion

Three different criteria have been presented for detection of satellite maneuver and satellite orbit and clock failures. In this subsection, these are applied together for classification of BDS unhealthy states. The BDS ephemeris and satellite clock error correction are uploaded every hour for each satellite. However, sometimes the satellite will be in normal state at the beginning of the hour but will malfunction before the end of the hour. Under this case, two groups of ephemerides with different health flags can be found in the same hour in the recorded broadcast ephemeris file provided by the International GPS Service (IGS). Nevertheless, the other parameters in the ephemerides are usually the same. On the other hand, a wrong record may also lead to two groups of ephemeris with different health flags in one hour. Therefore, it is necessary to distinguish between these two situations. Since consecutive incorrect recording is not likely to happen, we only need to focus on the unhealthy state that lasts for less than one hour. Consequently, data preprocessing is required before BDS unhealthy state classification. A four-step classification method is presented, as follows:

1. Find the unhealthy flag in the broadcast ephemeris. If the state lasts for more than one hour, go to step 2. If not, go to step 4.
2. Choose appropriate thresholds for mspi, uspi, and usci, namely th1, th2, and th3, respectively. Calculate the uspi and usci according to Equations (2) and (3), respectively. If the uspi is larger than th2 and the usci is less than th3, the unhealthy state corresponds to an excessive orbit error. If the uspi is less than th2 and the usci is more than th2, the unhealthy state corresponds to an excessive clock error. If both the uspi and usci are larger than the thresholds, the unhealthy state is most likely caused by a satellite payload failure.
3. If both the uspi and usci are less than the thresholds, then calculate the mspi according to Equation (1). If the mspi is larger than the threshold, the unhealthy state is caused by a satellite maneuver. If not, the unhealthy state is caused by an inbound or outbound satellite.
4. Since the satellite unhealthy state lasts for less than one hour, the unhealthy state is definitely not caused by a maneuver. If both the uspi and usci are less than the thresholds, the unhealthy state is very likely to be an incorrect recording. However, if at least one of them is larger than the threshold, the unhealthy state can be judged as having been caused by the corresponding anomaly.

## 4. Unhealthy State Statistics from July 2017 to June 2018

One-year broadcast ephemeris files from July 1, 2017 to June 30, 2018 are analyzed to validate the above analyses. Based on empirical data, the thresholds for mspi, uspi, and usci were set to 500, 10, and 10 m, respectively. The characteristics of the unhealthy states were highly related to the satellite types; therefore, only the results of C03, C07, and C11 are provided here to represent the GEO, IGSO, and MEO satellites, respectively. At first, the unhealthy states that lasted for more than one hour are shown. Afterward, the unhealthy states that lasted for less than one hour will be accounted for.

### 4.1. GEO Unhealthy State Statistics

**Figure 3:**
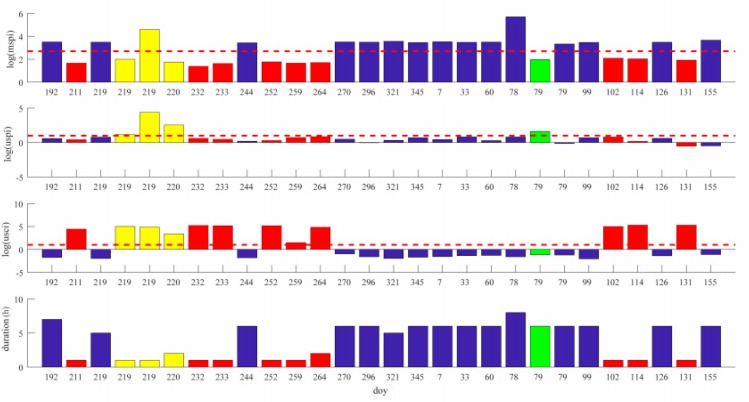
Unhealthy state statistics of C03. The subfigures correspond to mspi, uspi, usci, and duration of the unhealthy states, respectively. Blue bars represent Type 1 unhealthy states. Green bars represent Type 2 unhealthy states. Red bars represent Type 3 unhealthy states. Yellow bars represent Type 4 unhealthy states.

The mspi, uspi, and usci, and the duration of the unhealthy states of BDS GEO satellite C03 are shown in Figure 3. The horizontal axis represents the day of year (doy). Bars with different colors represent different unhealthy types. The blue bars represent an unhealthy state caused by a satellite maneuver, the red bars represent an unhealthy state corresponding to excessive satellite clock error, the green bars represent an unhealthy state corresponding to excessive satellite orbit error, and the yellow bars represent an unhealthy state corresponding to both excessive orbital and clock errors.

It can be seen that there were 15 orbital maneuvers for C03 in one year, and most of them lasted for about six hours. The uspi and usci values for the corresponding unhealthy states were all below the threshold, indicating that there were no orbital or clock anomalies. All the other unhealthy states can be accounted for by excessive orbital or clock error. Since the C03 is a GEO satellite, whose orbit is relatively stationary, there were no inbound or outbound cases. Figure 3 also shows that most of the unhealthy states corresponding to orbital or clock error were fixed in one or two hours, much shorter than the unhealthy duration caused by a maneuver.

### 4.2. IGSO Unhealthy State Statistics

**Figure 4:**
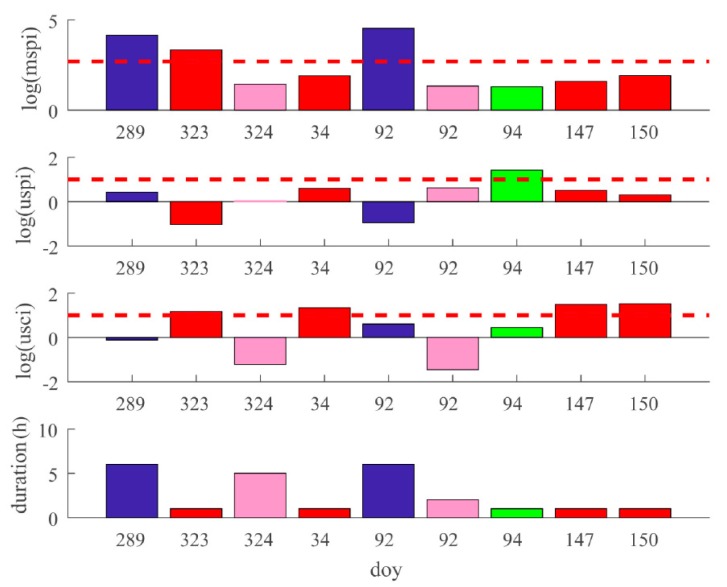
Unhealthy state statistics of C07. The subfigures correspond to mspi, uspi, usci, and duration of the unhealthy states, respectively. Blue bars represent Type 1 unhealthy states. Green bars represent Type 2 unhealthy states. Red bars represent Type 3 unhealthy states. Pink bars represent Type 5 unhealthy states.

The mspi, uspi, and usci, and the duration of the unhealthy states of BDS IGSO satellite C07 are shown in Figure 4. Unlike the GEO case, there were only two satellite maneuvers, each lasting for six hours. It should be noted that there were some unhealthy states whose mspi, uspi, and usci values were all under the thresholds, which are represented by pink bars. These states corresponded to the satellite inbound or outbound cases. It can be seen that such states were all after satellite anomalies or maneuvers, because the old observation data can no longer be used after an anomaly. Therefore, the accuracy of the predicted satellite orbital results may deteriorate rapidly after the satellite leaves the view of the monitor stations. Consequently, the broadcast ephemeris will not reflect accurate satellite position when the satellite returns to the visible area. To avoid such a phenomenon, the uploading station of the BDS sets the satellite to be unhealthy before the satellite leaves the view of the stations. It should be noted that the reason for the unhealthy states (corresponding to the pink bars) cannot be determined from the figure. The details of such unhealthy states will be depicted and analyzed thoroughly in Section 4.4.2.

### 4.3. MEO Unhealthy State Statistics

**Figure 5:**
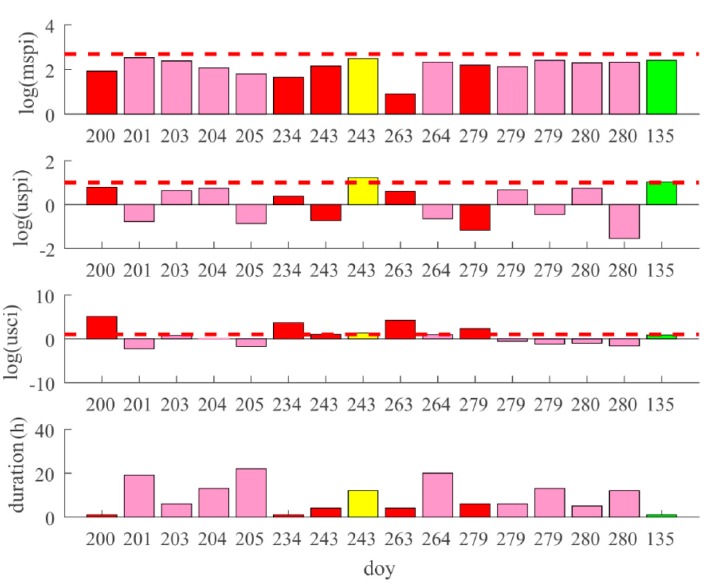
Unhealthy state statistics of C011. The subfigures correspond to mspi, uspi, usci, and duration of the unhealthy states, respectively. Green bars represent Type 2 unhealthy states. Red bars represent Type 3 unhealthy states. Yellow bars represent Type 4 unhealthy states. Pink bars represent Type 5 unhealthy states.

The mspi, uspi, and usci, and the duration of the unhealthy states of BDS MEO satellite C11 are shown in Figure 5. There was no maneuver for C11 in this year. Compared with the GEO and IGSO cases, there are more pink bars. That is because an MEO satellite runs all over the world; however, most of the monitor stations are located in China. Therefore, there are many more satellite inbound and outbound cases. Similar to Figure 4, the unhealthy states due to inbound or outbound satellites might occur after anomalies, but there were more such states. For example, there was an anomaly on doy 200. After that, there were consecutive pink bars on doy 201, 203, 204, and 205. The more frequent unhealthy states were probably because it was harder for the BDS stations to accumulate observation data due to the more frequent departures and entries of the MEO satellites.

### 4.4. Characteristics of Unhealthy States

According to the analysis in the previous chapter, there is a wide variety of unhealthy states for the BDS satellites, and their performance varies. Through careful analysis and research, many interesting rules can be summarized. These rules will be introduced in this subsection.

#### 4.4.1. North–South and East–West Station-Keeping

**Figure 6:**
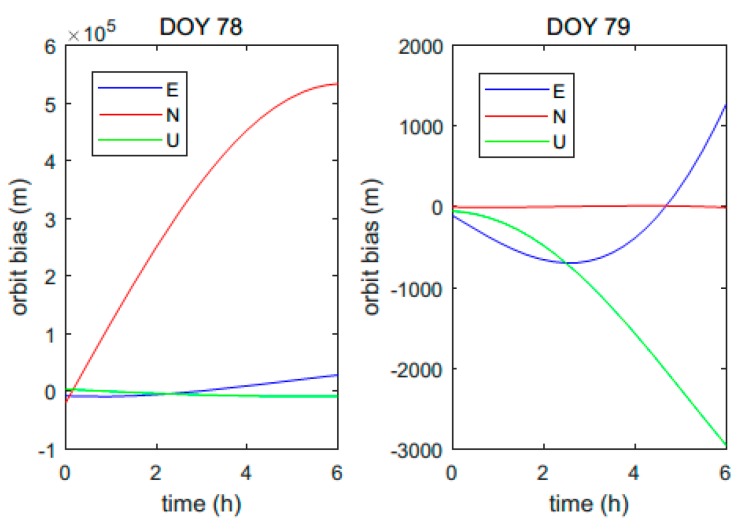
Orbit biases of east, north, and up directions between orbit results calculated with ephemerides before and after maneuvers of C03. Left and right subfigures correspond to results of doy 78 and doy 79, respectively.

It is explicitly shown in Figure 3 that the mspi of the maneuver on doy 78 was much larger than those on the other days. According to the physical meaning of mspi, the satellite orbit change was much larger in the maneuver on doy 78. In fact, this maneuver was a so-called north–south station-keeping maneuver, and the other maneuvers that induced much fewer orbit changes were east–west station-keeping ones. In order to view the difference between these two types of maneuvers, the orbit differences in the East, North, Up (ENU) coordinate system calculated with ephemerides before and after the maneuvers of C03 on doy 78 and 79, respectively, are shown in Figure 6.

As can be seen, the orbit change in the north direction of the north–south maneuver was over 5 × 10^5^ m, which was much greater than the changes in the east and up directions. On the other hand, the orbit change in the north direction in the east–west maneuver on doy 79 was only several meters, which was much smaller compared with the changes in other directions.

It should be noted that even though all the orbit changes in a period of time are shown in the figure, only the changes after the satellites finished the maneuver had clear physical meaning, because the healthy ephemerides before and after maneuvers could not reflect the precise satellite orbit when maneuvers were being performed.

In addition to the C03, consecutive north–south and east–west maneuvers were detected on doy 53 and 54 in 2018 for C01, doy 324 and 325 in 2017 for C02, doy 29 and 30 in 2018 for C04, doy 282 and 283 in 2017, and doy 134 and 135 in 2018 for C05. It can be concluded from these statistics that there will definitely be an east–west maneuver on the second day of a north–south maneuver for the BDS GEO satellites. There are one or two north–south maneuvers in one year for each GEO satellite, which is more frequent than the results reported in [3,12].

A north–south maneuver needs to change the orbital plane to compensate for the effect of lunar/solar gravitation. However, in an east–west maneuver, thruster burns tangential to the orbit to control the orbital period and eccentricity [3]. As a result, a north–south maneuver needs much more fuel than an east–west one and it usually has more influence on satellite position, as shown in Figure 6. For the BDS GEO satellites, an east–west maneuver is always performed to adjust the orbit after a north–south maneuver.

#### 4.4.2. Validation of Unhealthy States Due to Inbound or Outbound Satellites

**Figure 7:**
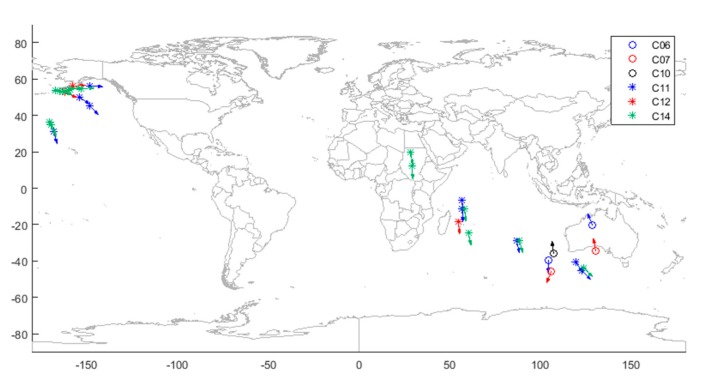
Satellite position and direction of motion corresponding to unhealthy states caused by satellite departure or entry.

There are several unhealthy states represented by pink bars whose mspi, uspi, and usci values are all below the thresholds. To interpret why these satellites were set as unhealthy, the sub-satellite points (points at which lines between the satellites and the center of the Earth intersect the Earth’s surface) of the corresponding satellites when the broadcast ephemerides were set to unhealthy and the instantaneous velocity directions are shown in Figure 7. It can be seen that all the sub-satellite points were far away from the mainland of China and most of the satellites were moving away. Only three satellites were inbound, all of which were IGSO satellites, and their unhealthy time only lasted for one to two hours, which means that the satellite orbit and clock error were below the threshold again after the satellites returned to the visible region of the monitor stations. Taking Beijing as the reference point and calculating all the elevation angles of the satellites, the average elevation angle of the satellites in Figure 7 is 10.26°. The average elevation angle can prove that all the pink bars are caused by inbound or outbound satellites.

#### 4.4.3. Unhealthy States that Last for Less Than One Hour

**Figure 8:**
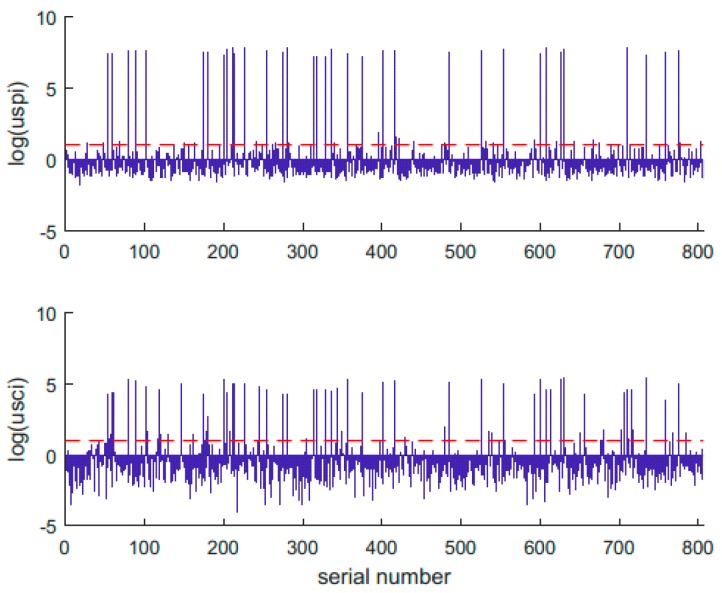
Values of unhealthy satellite positioning index (uspi) and unhealthy satellite clock index (usci) corresponding to unhealthy states that lasted for less than one hour.

In the broadcast ephemeris, there are many unhealthy states that last for less than one hour. As a result, there may be more than one group of ephemerides of one satellite at a particular epoch. Most of them may be incorrect records, but some are caused by poor accuracy of orbit and clock prediction or payload failure. Therefore, the parameters of uspi and usci can be applied to distinguish an incorrect record from an anomaly. All the uspi and usci values of the unhealthy states that lasted for only one hour from July 2017 to June 2018 are shown in Figure 8.

**Figure 9:**
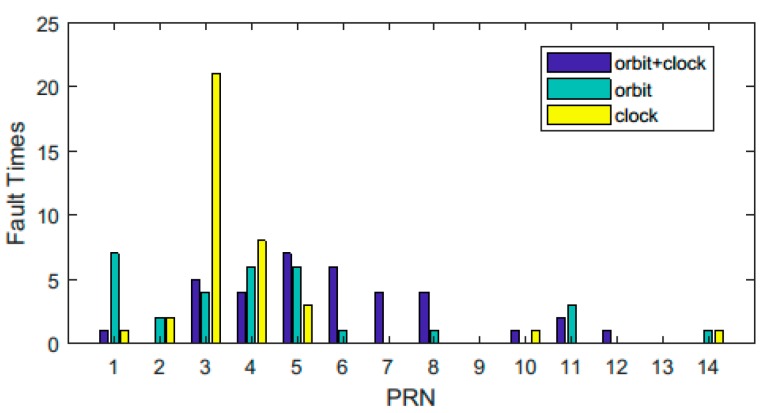
Anomaly times of different satellites corresponding to unhealthy states that lasted for only one hour. Different colors refer to different types of faults.

Figure 8 shows that there were more than 800 unhealthy states that lasted for less than one hour in the ephemeris files in the one-year time. Most of the uspi and usci values were less than the threshold. Some of these unhealthy states may have been caused by inbound or outbound satellites. The others may have been incorrectly recorded, and the satellites would not affect the BDS service if they were not set as unhealthy. The other uspi and usci values of the unhealthy states were larger than the threshold; these states corresponded to actual anomalies that can deteriorate the system service performance seriously if they are not set as unhealthy. The correspondence between these states and the satellite pseudo-random noise (PRN) is shown in Figure 9.

It can be seen that there were many more anomalies concentrated on the GEO satellites and the clock-related problems of the C03 satellite than the other problems.

#### 4.4.4. Overall Statistics of BDS Unhealthy States

Table 1 shows the occurrence of different types of unhealthy states for different satellites in one year. As can be seen, there were 287 unhealthy states caused by satellite maneuvers, anomalies, and inbound or outbound satellites. About 24.7% of the unhealthy states were due to satellite orbital maneuvers, and 15% corresponded to excessive orbital error. Type 3 and type 4 accounted for 32.1% and 17.1% of the total unhealthy states, respectively. In other words, about half of the unhealthy states were related to excessive clock error, which may be caused by satellite clock or payload failure. In addition, about 11.1% of unhealthy states were classified as type 5, and all of them corresponded to IGSO and MEO satellites. Since the type 5 unhealthy states that lasted for less than one hour are not recorded here, there were more actual type 5 states than the number shown in Table 1.

There were 71 orbital maneuvers from July 2017 to June 2018. The maneuver frequency of the GEO satellites was much higher than that of the other two types of satellites. Each IGSO satellite had two maneuvers, and no MEO satellite maneuvers were detected. Each GEO satellite had 6 to 15 maneuvers a year. C03 had the most and C04 had the fewest maneuvers. All phenomena were consistent with the results in [3].

Table 1 also shows that the BeiDou GEO satellites had much more nonmaneuvering anomalies than the IGSO and MEO satellites. C03, C04, and C05 had the most nonmaneuvering anomalies, indicating that these satellites were less reliable compared with the others.

Table 2 shows the statistics of different types of the unhealthy states that lasted for more than one hour. As can be seen, both the uspi and usci values of type 1 unhealthy states corresponding to the maneuvers were less than the threshold, and the average duration was 6.21 hours. The usci values of the type 2 unhealthy states were all lower and the uspi values were higher than the threshold; however, they were only dozens of meters. The positioning accuracy of the BDS user would not be affected severely if the measurements of these satellites were used. In other words, the type 2 unhealthy states were not very serious. As for the type 3 and type 4 unhealthy states, most of the usci values were much larger than the threshold, indicating that the unhealthy states corresponding to the satellite clock were very hazardous. The average duration was about two hours for types 2, 3, and 4, which means that these nonmaneuver anomalies were usually solved very quickly and the corresponding unhealthy satellites could be available in a very short time after the anomalies occurred.

Table 3 shows the statistics of the different types for the unhealthy states lasting less than one hour. The duration of all unhealthy states due to maneuvers is more than one hour; therefore, there is no type 1 in Table 3. Similar to the results in Table 2, the uspi values of type 2 were not very large even though they all exceeded the threshold. However, the usci values of type 3 and 4 were usually much larger than the threshold. Table 2 and Table 3 show that the unhealthy states with different durations had similar characteristics. The anomalies related to the satellite clock were usually more serious than the ones only related to excessive orbit error.

## 5. Impacts of Unhealthy States on System Performance

As we know, positioning accuracy is highly related to the user-equivalent range error (UERE) and position dilution of precision (PDOP). The standard deviation of the positioning error can be given as follows [19]:

(7) $$\sigma_{P}=PDOP\cdot \sigma_{UERE}$$

When some of the satellites are unhealthy, the geometry of the user/satellites will be changed and the PDOP will increase. Consequently, the positioning accuracy will deteriorate. Since the $\sigma_{UERE}$ of the satellites is relatively constant, the most important factor that affects the system performance is the PDOP increment due to the unhealthy satellites [20].

**Figure 10:**
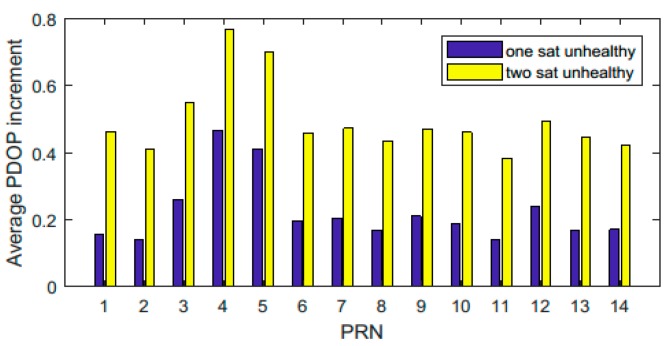
Average position dilution of precision (PDOP) increments in the BDS service area when one or two satellites were unhealthy in January 2018.

Statistics of the broadcast ephemeris files from July 2017 to June 2018 show that there were one, two, and three unhealthy satellites 10.94%, 0.6%, and 0.03% of the time, respectively. That is to say, the number of unhealthy satellites was no more than two most of the time. Therefore, only the cases with one or two unhealthy satellites are considered here. The average PDOP increments in the service area when different satellites were unhealthy in January 2018 are provided in Figure 10. The blue bars represent results when only one satellite was unhealthy. The yellow bars represent the average increments when one certain satellite and another satellite were unhealthy simultaneously. It can be seen that the PDOP increments were from 0.15 to 0.5 when only one satellite was unhealthy, and from 0.3 to 0.8 when two satellites were unhealthy. Since the average $\sigma_{UERE}$ is about 1.5 meters for the BDS [21], the average decrease of positioning accuracy is about 0.2 to 0.75 meters for one unhealthy satellite and 0.45 to 1.2 meters for two unhealthy satellites.

**Figure 11:**
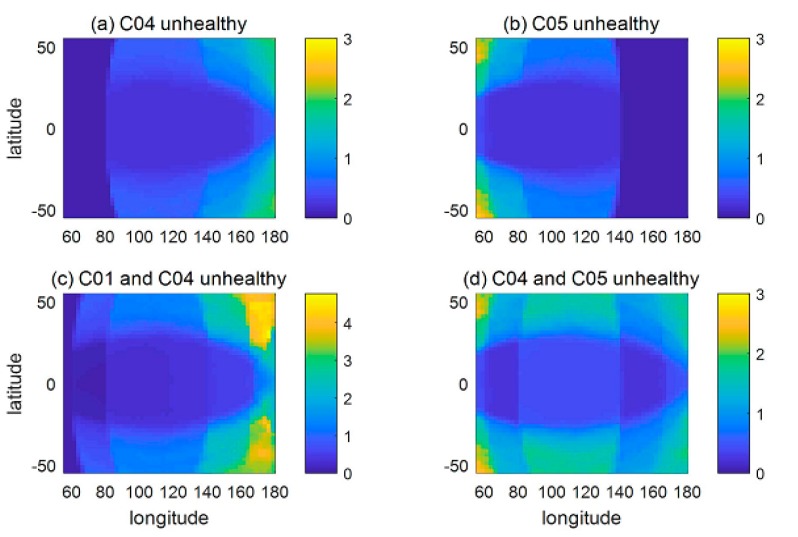
PDOP increments in the service area.

To evaluate the PDOP increments in the overall system service area, the PDOP increments when one or two satellites were unhealthy are shown in Figure 11. The outline of each subfigure corresponds to the service area of the BDS, which is from 55° S to 55° N, 55° E to 180° E. Figure 11a,b shows the results when one satellite was unhealthy. C04 and C05 are chosen here, corresponding to the cases whose PDOP increments were the largest and second largest when one satellite was unhealthy, as shown in Figure 10. Figure 11c,d shows the results when two satellites were unhealthy. Satellite pairs (C01, C04) and (C04, C05) are chosen here, corresponding to the cases whose PDOP increments were the largest and second largest when two satellites were unhealthy. 

In all the cases, the average PDOP increments in the corners of the service area were the largest, and increments in the important service area of the BDS, which is from 5° N to 55° N, 70° E to 145° E, were relatively small since most of the BDS satellites were above this area. The largest average PDOP increment was about five, which occurred near longitude 180° when C01 and C04 were unhealthy, since the six IGSO satellites were located at two orbits whose recursive longitudes are 95° E and 118° E, respectively. The distance between the IGSO satellites and the east margin of the service area is farther than between the IGSO satellites and the west margin. Therefore, the largest PDOP increments occurred when the C01 (140° E) and C04 (160° E) were unhealthy simultaneously due to satellites being less visible and bad satellite/user geometry near longitude 180°.

It should be noted that the PDOP increments shown in Figure 11 only represent the average situation. Since the geometry of healthy and unhealthy satellites changes over time, the PDOP increments may be different from the results in the figure. However, since most of the satellites in the current BDS are GEO and IGSO satellites, the geometry will not change dramatically and the results in Figure 11 can be used as representative of the PDOP changes when one or two satellites are not available.

## 6. Discussion and Conclusions

In this study, BDS satellite unhealthy states are categorized into five types based on the broadcast ephemeris. Three criteria, namely maneuver satellite positioning index (mspi), unhealthy satellite positioning index (uspi), and unhealthy satellite clock index (usci), are proposed for unhealthy state classification. The following interesting phenomena are found by analyzing the unhealthy state statistics from July 2017 to June 2018. First, there was always an east–west station-keeping maneuver on the second day of a north–south one, which made the satellite unavailable for two consecutive days. More attention should be paid to the performance of the system during these periods. Second, the unhealthy states in which all criteria were below the thresholds were due to inbound or outbound satellites. This is because of insufficient monitor stations out of the border of China and a lack of observation data when the IGSO or MEO satellites left the visible area of the Chinese mainland, which may result in a large error in orbit or clock prediction. This problem can be solved by establishing more monitor stations around the world. Third, there were a lot of unhealthy states that lasted for less than one hour in the broadcast ephemeris, but only a small part was caused by satellite anomalies, which may affect system performance. Fourth, about 24.7% of unhealthy states were caused by satellite maneuvers and the corresponding average unhealthy duration was about 6.2 h. The average duration of the unhealthy states related to excessive satellite orbit or clock error was about two hours. Compared with the unhealthy states only related to orbit, those related to the satellite clock were usually more hazardous since they usually induced much larger biases. In addition, statistics show that the number of unhealthy satellites was usually no more than two at a certain epoch. The performance changes when one or two BDS satellites are unhealthy were simulated. Results show that the performance of the important service area of the system is less affected, and the margin of the system service area is more affected. Considering the current UERE of the BDS, when one or two satellites are unhealthy, the average positioning accuracy in the service area will decrease by less than 0.75 and 1.2 m, respectively.

## Figures and Tables

**Table 1:** Occurrence of different types of unhealthy states for different satellites.

PRN	SAT	Type 1	Type 2	Type 3	Type 4	Type 5	Sum
C01	GEO	14	7	1	1	0	23
C02	GEO	11	2	5	0	0	18
C03	GEO	15	6	29	8	0	58
C04	GEO	6	9	24	6	0	45
C05	GEO	13	7	10	10	0	40
C06	IGSO	2	2	4	8	2	18
C07	IGSO	2	1	4	4	3	14
C08	IGSO	2	3	1	4	0	10
C09	IGSO	2	1	1	0	0	4
C10	IGSO	2	0	1	3	1	7
C13	IGSO	2	3	0	2	0	7
C11	MEO	0	1	5	2	9	17
C12	MEO	0	0	0	0	3	3
C14	MEO	0	1	7	1	14	23
Sum		71	43	92	49	32	287
Proportion		24.7%	15.0%	32.1%	17.1%	11.1%	100%

**Table 2:** Statistics of different types of unhealthy states lasting more than one hour.

	Type 1		Type 2		Type 3		Type 4	
Metric	uspi	usci	uspi	usci	uspi	usci	uspi	usci
Average (m)	2.72	0.76	22.63	2.63	3.15	5.19 × 10^4^	9.89 × 10^3^	8.03 × 10^4^
Maximum (m)	8.26	6.62	51.81	7.69	9.35	2.85 × 10^5^	9.34 × 10^4^	2.53 × 10^5^
Minimum (m)	0.05	0.005	10.67	0.08	0.04	19.81	10.21	21.12
Times	71		12		55		14	
Average Duration (h)	6.21		2.09		2.16		2.08	

**Table 3:** Statistics of different types for unhealthy states lasting less than one hour.

	Type 2		Type 3		Type 4	
Metric	uspi	usci	uspi	usci	uspi	usci
Average (m)	16.90	0.28	1.28	1.07 × 10^4^	3.69 × 10^7^	9.42 × 10^4^
Maximum (m)	75.24	2.12	6.42	1.08 × 10^5^	6.53 × 10^7^	2.82 × 10^5^
Minimum (m)	10.18	0.02	0.07	10.04	23.79	549.85
Times	31		37		35	
Average Duration (h)	<1		<1		<1	

## Abbreviations

MEO	Medium Earth Orbit
GEO	Geostationary Orbit
IGSO	Inclined Geosynchronous Orbit
BDS	BeiDou System
GPS	Global Positioning System
SIS	Signal-in-space
URE	User Range Error
RAIM	Receiver Autonomous Integrity Monitoring
SRP	Solar Radiation Pressure
RMS	Root Mean Square
PRN	Pseudo-Random Noise
UERE	User-Equivalent Range Error
PDOP	Position Dilution of Precision
